# Luminescence from Zinc Oxide Nanostructures and Polymers and their Hybrid Devices

**DOI:** 10.3390/ma3042643

**Published:** 2010-04-12

**Authors:** Magnus Willander, Omer Nur, Jamil Rana Sadaf, Muhammad Israr Qadir, Saima Zaman, Ahmed Zainelabdin, Nargis Bano, Ijaz Hussain

**Affiliations:** Department of Science and Technology (ITN), Campus Norrköping, Linköping University, SE 60174 Norrköping, Sweden ; E-Mails: omeno@itn.liu.se (O.N.); sadra@itn.liu.se (J.R.S.); muhqa@itn.liu.se (M.Q.I.); saiza@itn.liu.se (S.Z.); ahmza@itn.liu.se (A.Z.); narba@itn.liu.se (N.B.); ijaz.hussain@ecospark.se (I.H.)

**Keywords:** ZnO nanostructures, deep center luminescence, light emitting diodes, hybrid technology

## Abstract

Zinc oxide (ZnO) is a strong luminescent material, as are several polymers. These two materials have distinct drawbacks and advantages, and they can be combined to form nanostructures with many important applications, e.g., large-area white lighting. This paper discusses the origin of visible emission centers in ZnO nanorods grown with different approaches. White light emitting diodes (LEDs) were fabricated by combining n-ZnO nanorods and hollow nanotubes with different p-type materials to form heterojunctions. The p-type component of the hybrids includes p-SiC, p-GaN, and polymers. We conclude by analyzing the electroluminescence of the different light emitting diodes we fabricated. The observed optical, electrical, and electro-optical characteristics of these LEDs are discussed with an emphasis on the deep level centers that cause the emission.

## 1. Introduction

Zinc oxide (ZnO), a II-VI direct wide bandgap semiconductor, has been studied by the scientific community since the 1930s [1]. Although it has unique and interesting properties, such as a relatively high exciton binding energy (60 meV), and a wide bandgap (3.34 eV), and is piezoelectric, biologically safe and biocompatible [2], researchers’ work with ZnO has previously been focused on obtaining stable p-type dopants for ZnO. In addition to these excellent properties, ZnO possesses a large number of extrinsic and intrinsic deep-level impurities and complexes (clusters) that emit light of different colors [2], including violet, blue, green, yellow, orange and red, *i.e.*, all constituents of white light [2,3,4]. Because of this, ZnO is considered to be attractive for applications requiring luminescent materials. ZnO, especially in its nanostructure form, is currently attracting intense global interest for photonic applications [2]. ZnO has the additional advantages of being easy to grow and possessing the richest known family of nanostructures [5]. The present global interest in ZnO nanostructures is motivated by the possibility of growing them on any p-type substrate and hence producing high quality pn heterojunctions [2]. The interest in optoelectronic applications arises from the possibility of developing low energy and environmentally friendly white light emitting technologies and laser diodes that operate above room temperature [2]. The renewed interest in utilizing the excellent properties of ZnO in optoelectronic devices is mainly due to the ZnO ambipolar doping problem mentioned above. This problem frequently occurs in wideband gap materials, in which it is very easy to dope the material with one polarity but is very difficult to dope the same material with the other polarity [6]. As ZnO is naturally n-type, it is very difficult to dope it with materials of p-type polarity [6]. Several laboratories have reported p-type ZnO, but their results were difficult to reproduce in other laboratories and hence remain controversial. Elements from group I, including Li, Na, and K, as well as elements like Cu and Ag, are supposed to be good acceptors when replacing a Zn site, and they form deep acceptors with ionization energies around a few hundred meV above the valence band [7]. This implies that under normal conditions, *i.e.*, at equilibrium, doping can be achieved without any ionization leading to free holes. Moreover, at high levels of doping with such elements, interstitial Li (or Ag) atoms will act as donors and compensate many acceptors [7,8,9]. Another possibility for doping ZnO to p-type is to use elements from group V on the O site, including N, P, Sb, and As. Nevertheless, most efforts to use these elements have led to poorly reproducible results. An elegant summary of all of these efforts is documented in Look *et al.* [10]. Most recently, there have been successful reports of doping ZnO with N, forming a level with ionization energy of around 100 meV, less than the 160 meV ionization energy of the standard Mg acceptor in GaN [6]. Nevertheless, due to the existence of other native deep levels close to the conduction band, the compensation effect makes these efforts unsuccessful in producing stable and highly doped p-type ZnO materials.

The difficulty in doping ZnO to p-type polarity has led researchers to seek to create heterojunctions with other p-type semiconductor materials to enable ZnO to be used in optoelectronic devices. These efforts began by growing n-type ZnO thin films on p-type substrates. However, due to lattice mismatches, most of these efforts have not led to the development of device-quality heterojunctions. The efforts in growing thin films of n-type ZnO on different p-type substrates, along with many of the fundamental properties of ZnO, are described in the comprehensive review written by Özgur *et al.* [11]. Nano-structures, especially nanorods or nanowires, possess a relatively large surface area to volume ratio, enabling them to release stress and strain due to lattice mismatch with other materials. In addition, ZnO has been shown to be able to produce a rich family of different nanostructures; as a wurtzite structure, ZnO has a total of 13 different facet growth directions: <0 0 0 1>, <0 1 −1 0>, <2 −1 −1 0>. Together with a pair of polar surfaces {0001}, this uniquely structured material has been demonstrated to form a diverse group of nanostructures: nanorods, nanobelts, nanocombs, nanosprings, nanorings, nanobows, nanojunction arrays, and nanopropeller arrays, which are formed largely due to the highly ionic character of the polar surfaces [12]. Some ZnO nanostructures (namely tetrapods) were unintentionally synthesized as early as 1944 [13]. At that time, there were no microscopes with sufficient resolution to view the synthesized structures, which have since been identified as tetrapods [13]. The different growth methods used to obtain ZnO nanostructures can be divided into two main groups: low (<100 °C) and high (up to 1000 °C) temperature techniques. Willander *et al.* provide a thorough review of these different growth techniques; the reader is directed to [2]. High quality ZnO nanostructures have been grown on a variety of crystalline as well as amorphous (polymer) substrates and formed excellent pn heterojunctions, in contrast to thin films of ZnO, which have shown very limited success in forming heterojunctions. One advantage of n-ZnO nanorods on any p-substrate is that each nanorod will form a discrete, separated pn junction, and hence a large-area light emitting diode can be designed without compromising the junction area, which would lead to large reverse leakage currents. This is an important property that is advantageous for large-area lighting commercialization.

This paper discusses the different native point defects in ZnO together with their relevant luminescent properties. The origin of the different deep-level emission bands usually observed in ZnO is briefly reviewed. This discussion is followed by examples of recently fabricated light emitting diodes (LEDs) based on ZnO nanorods and nanotubes, fabricated using different crystalline and amorphous p-type substrates. The electrical, optical and electro-optical characteristics of these visible light emitting diodes will all be discussed in connection to the deep-level defects present in different ZnO nanorods and nanotubes grown by high or low temperature techniques. We will present results from LEDs grown by low temperature chemical growth at temperatures as low as 50 °C on plastic flexible substrates coated with p-type polymers suitable for large area lighting.

## 2. Luminescent Centers in ZnO

Efficient donors and acceptors have energy levels near the conduction and valence bands, respectively; deep centers also exist with energy levels deep in the forbidden gap. The room temperature photoluminescence (PL) spectrum of ZnO nanorods/nanowires with diameters larger than 20 nm is similar to the PL spectra of bulk ZnO. This room temperature PL spectrum is normally characterized by near-band-edge (NBE) ultra-violet (UV) emission and at least one broad band emission due to deep levels, called DLE. DLE refers to the broad band extending from just above 400 nm up to 750 nm, *i.e.*, the whole visible spectrum. The broadness of the band results from the fact that it represents a superposition of many different deep levels emitting at the same time. Different reports have suggested different deep levels as the origin of the observed emissions. Before discussing the origin of the deep band emissions, it is important to discuss most of the known deep levels in ZnO and some of their important properties such as their formation energy and contribution to conductivity. Although no consensus exists on the origin of the broad deep band emission, the broad nature of the emission suggests the possibility that it is a combination of many emissions. The deep levels of ZnO are divided into extrinsic and intrinsic deep levels.

The possible intrinsic ‘native’ deep levels in ZnO are oxygen vacancy (V_O_), zinc vacancy (V_Zn_), oxygen interstitial (O_i_), zinc interstitial (Zn_i_), oxygen anti-site (O_Zn_), and zinc anti-site (Zn_O_). This is in addition to native defect clusters, which are usually formed by the combination of two point defects or one point defect and one extrinsic element, e.g., a V_O_Zn_i_ cluster formed by Zn_i_ and V_O_. This V_O_Zn_i_ cluster is one of the clusters that has been previously identified and is situated 2.16 eV below the conduction band minimum. These native point defects often directly or indirectly control doping, compensation, minority carrier lifetime and luminescence efficiency in semiconductors [14]. Native defects are often invoked to explain the fact that ZnO always exhibits a high level of unintentional n-type conductivity. Even the difficulty in obtaining stable p-type doping is closely related to a compensation effect connected to intrinsic native defects that lie in the forbidden gap (deep centers). We will review the basic properties of these different native defects below. The following discussion is mainly based on results obtained using a comprehensive, first-principles investigation of native point defects in ZnO using density functional theory within the local density approximation [14]. The concentration of a point defect depends on its formation energy. At thermodynamic equilibrium and in dilute cases (no defect-defect interaction), the concentration of a point defect (c) is given by [14]:

(1) $$c=N_{sites}\exp\left(-\frac{E^{f}}{k_{B}T}\right)$$

where c is the point defect concentration, $E^{f}$ is the formation energy, $N_{Sites}$ is the number of available sites to accommodate the defect, k_B_ is Boltzmann’s constant, and T represents temperature. According to equation 1, defects with high formation energies will occur at low concentrations. The formation energy $E^{f}$ of point defects is not constant, but rather depends on the growth parameters and annealing conditions [15]. The formation energy of an oxygen vacancy depends on the abundance of oxygen and zinc atoms in the growth environment. Furthermore, if the vacancy is charged then the formation energy depends on the Fermi level (E_F_), *i.e.*, the electron chemical potential. The chemical potential depends on the growth conditions, which can either be oxygen-rich, zinc-rich or in between these two extremes. Hence, the chemical potential is usually treated as a variable and is chosen according to certain rules. In reality, the growth environment controls the concentration of native defects in ZnO. For further details on the limitations of chemical potential values, the reader is advised to Janotti *et al.* and van de Walle *et al.* [14,15]. As discussed above, these deep levels introduce levels in the bandgap of the semiconductor that involve transitions between different charge states. The transition levels can be experimentally observed when the final charge state fully relaxes to its equilibrium configuration after the transition, such as in deep level transient spectroscopy (DLTS) [16]. Conventionally, if the transition level is situated such that the defect is most likely to be ionized at room temperature or at the device operating temperature, then this is called a shallow transition level [14]. If the transition level is unlikely to be ionized at room temperature, then it is a deep transition level. The first step in the discussion on deep level native defects in ZnO is to consider V_O_. V_O_ and Zn_i_ have long been suggested to be sources of the observed unintentional doping in ZnO, which is due to shallow levels situated 30–40 meV below the conduction band minima [17,18]. The assignment of V_O_ or Zn_i_ to the unintentional n-type doping originated from the fact that the growth of ZnO crystals was typically performed in a Zn-rich environment, and hence the dominant native defects were assumed to be V_O_ and Zn_i_. Nevertheless, recent careful theoretical study revealed that this claim was incorrect for both V_O_ and Zn_i_, as will be discussed below [14]. The formation energy of V_O_ was found to be quite high in n-ZnO material, even under extreme conditions, where it has a value of 3.27 eV. According to equation 1, V_O_ will always occur in low concentrations under equilibrium conditions, and it is not expected to be the source of the unintentional n-type doping. According to the energy calculations, isolated V_O_ cannot be the source of electrons in the conduction band in ZnO. In fact, in p-type doped ZnO, V_O_ assumes a 2+ charge state and hence provides a potential source of compensation in p-type ZnO [14]. This theoretical investigation [14] was consistent with experimental evidence from positron annihilation spectroscopy studies [19,20] that studied grown and electron-irradiated ZnO samples. It has been shown experimentally that the dominant defect in electron-irradiated n-ZnO samples is V_Zn_, with the Fermi level located 0.2 eV below the conduction band minima [14,19,20]. Neutral V_O_ was also detected in these experiments. These results imply that charged V_O_, if present, will only be in low concentrations below the detection limit due to their high formation energy as discussed above. Nevertheless, other experimental measurements have shown that native defects, and especially V_O_ deep level defects, can contribute to the unintentional n-type conductivity of ZnO when present as complexes, but not as isolated native point defects [21]. On the other hand, V_Zn_ has the lowest formation energy of all of the native defects in n-type ZnO, while its formation energy in p-type ZnO is quite high [14]. This energy is low enough for $V_{Zn}^{2-}$ to occur in modestly doped ZnO and to act as a compensating center. Zinc vacancies usually introduce partially occupied states in the bandgap. These states are derived from the broken bonds of the oxygen’s nearest four neighbors and lie close to the valence band minima. These states are partially filled and can accommodate an electron, causing V_Zn_ to act as an acceptor. However, quantitative calculations showed that V_Zn_ levels are deep acceptors. On the other hand, zinc vacancies are not believed to contribute to the p-type doping of ZnO due to the high formation energy of V_Zn_ in p-type ZnO [14]. V_Zn_ has been observed in many as-grown n-ZnO materials and are more favorable when growth is performed in oxygen-rich conditions [14,19]. Zinc vacancies are situated 0.9 eV above the valence band minima, and hence a transition from the conduction band (or from a shallow donor) would yield a luminescence around 2.4 eV. This corresponds to the green luminescence observed in ZnO samples grown by many techniques, appearing at 2.4–2.5 eV. Hence, V_Zn_ is widely accepted to contribute to the broad band emission at this green wavelength, although V_O_ was also suggested as early as 1954 [22] to be the source of this green emission (see discussion below). On the other hand, for n-type ZnO, *i.e.*, for a Fermi level close to the conduction band, interstitial zinc has high formation energy even under Zn-rich conditions, with a formation energy that reaches 6 eV. This implies that under equilibrium conditions, Zn_i_ will be present in low concentrations and cannot contribute to the unintentional doping of ZnO. Moreover, the formation energy of $Zn_{i}^{2+}$ decreases rapidly as the Fermi level decreases toward the valence band minima. This implies that Zn_i_ is a potential source of compensation in p-type ZnO [14]. The excess of oxygen in the ZnO lattice can be accommodated through the existence of oxygen interstitials, which can exist in electrically active or inactive forms. Electrically active O_i_ occupies an octahedral site [14] and introduces states that can accept two electrons in the lower part of the ZnO bandgap. The result is a deep acceptor transition with states situated 0.72 eV and 1.59 eV above the valence band minima. The other form of O_i_ is an electrically inactive configuration, which has quite high formation energies for both forms of O_i_, except under extremely O-rich environments. This implies that O_i_ is not expected to be present in high concentrations under equilibrium conditions. The remaining native defects are anti-sites. Zinc anti-sites or oxygen anti-sites consist of zinc or oxygen atoms sitting at the wrong lattice position. All calculations have agreed that Zn_O_ forms shallow donors [23,24]. The final native defect is oxygen occupying an anti-site. Oxygen anti-sites can be created under non-equilibrium conditions, for example by irradiation or ion implantation [14]. Recent calculations indicated that O_Zn_ is a deep acceptor level with two possible transitions situated 1.52 eV and 1.77 eV above the valence band minima. All of the native defects discussed above can exist in different charged states or in a neutral state, and the formation of complexes between native defects and other extrinsic species in ZnO has also been reported. As mentioned above, most of these native defects introduce deep levels at different positions in the bandgap, and hence a rather large number of luminescence lines with different energies can be observed. This explains why all of the visible colors have been experimentally observed in different ZnO samples.

The main known extrinsic deep-level defects in ZnO are Li, Cu, Fe, Mn, and OH, each of which have been reported to emit at different wavelengths as discussed in more detail by Klingshirn *et al.* and Özgür *et al.* [6,11]. Different deep levels can produce different lines of the same color; one example of this is ZnO:Cu and ZnO:Co, which emit different green colors [6]. This phenomenon is an additional source of the discrepancy in explaining the observed emission of ZnO. Finally, hydrogen also plays an important role in the properties of the native defects. Hydrogen is not a deep level in ZnO, but we mention it due to its important role as a donor. Unlike other semiconductors where hydrogen can be positive or negative, hydrogen in ZnO is always positive (H^+^), *i.e.*, it acts as a donor and possesses low ionization energy [25].

**Figure 1:**
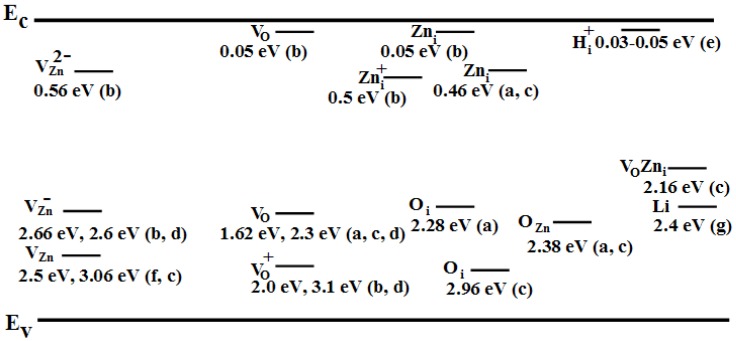
Energy levels of the different deep level defects in ZnO reported in the literature by different groups: (a) ref [54]. (b) ref. [43].(c) ref. [56]. (d) ref. [44]. (e) ref. [25]. (f) ref. [14] and (g) ref. [9,35]. The + and – symbols represent charged deep levels. Three shallow levels are also shown, due to neutral oxygen vacancies, positively charged extrinsic hydrogen, and neutral zinc interstitials. In addition, the position of the deep level due to V_O_Zn_i_ clusters is indicated.

As mentioned above, the origin of the deep level emission band (DLE) has been controversial for decades. Below, we will briefly discuss some of the different opinions about the origin of the DLE based on different findings. The common bands observed in ZnO are green luminescence, yellow luminescence, and red luminescence DLE bands [11]. The green luminescence band, which appears at energies of 2.4–2.5 eV, is the most thoroughly investigated DLE band in ZnO and has been the subject of the most debate. Several studies have been published regarding the origin of this band, and they have used different experimental setups and different samples grown under various conditions. The green luminescence has been observed in samples grown by a variety of techniques. There may be multiple sources of this luminescence because different transitions can lead to quite similar luminescent emission wavelengths. Zinc vacancies, one of the most probable native defects in ZnO, have been suggested by many authors to be the single source of this emission; see [26,27,28]. Oxygen vacancies have also been suggested by many authors [22,29,30,31]. In addition, zinc interstitials, oxygen interstitials, and other extrinsic deep levels including Cu have all been proposed as sources of the green luminescence emission in ZnO. For more details, the reader is directed to [2,6,11] and the references therein. More recently, the green emission band has been explained as originating from more than one deep level defect. In this recent investigation, V_O_ and V_Zn_, which have different optical characteristics, were both found to contribute to the broad green luminescence band [32,33,34]. The yellow emission band that appears at 2.2 eV was first observed in a Li-doped ZnO layer [9,35]. Li is located 0.8 eV above the valence band and constitutes a deep acceptor level in ZnO. Yellow emission has also been attributed to native deep level defects in ZnO, namely to oxygen interstitials [36,37]. The yellow emission band was also observed with metastable behavior in undoped bulk ZnO [11]. Under irradiation by a He-Cd laser, the green luminescence band mentioned above was gradually bleached, and yellow emission emerged and saturated with an excitation density of 10^-3^ W/cm^2^, implying that the associated deep level is present at a low density. The yellow emission band was recently observed in ZnO nanorods grown by low temperature (90 °C) chemical growth in different laboratories [38]. The origin of this band in these low-temperature grown samples was attributed to O_i_ or the presence of Li impurities in the initial growth material. A Zn(OH)_2_ group attached to the surface of ZnO nanorods grown by chemical methods has also been proposed as a possible source of the yellow deep-level defect emission band in these samples [39]. Yellow emission has been observed in many different grown ZnO nanorods, and it was demonstrated that the emission can be replaced by the green and red bands upon post-growth annealing [39]. This was explained by the fact that upon proper post-growth annealing, the hydroxyl group can desorbs and hence modify the emission from that of the as-grown ZnO nanorods [39]. Orange, orange-red and red emission bands have also been observed in ZnO [39]. The orange emission, which is not very common in ZnO, was proposed to be due to transitions related to oxygen interstitials [40], the orange-red emission was recently attributed to transitions associated with zinc vacancy complexes [41], and the red emission was proposed to be due to transitions associated with zinc interstitials [42]. Figure 1 shows a schematic diagram of the different energy levels (measured with respect to the conduction band edge) of the different deep-level defects reported by different groups. For completeness, the position of the extrinsic hydrogen energy level is also depicted, as this plays an important role in the n-type conductivity of ZnO. The energy of the V_O_Zn_i_ cluster is also indicated. Table 1 also summarizes some of the different colors observed in recent reports and their associated deep-level defects.

From the preceding discussion on the properties of the commonly reported deep level centers in ZnO and their associated possible transitions, it is clear that ZnO can emit luminescence over the entire visible region. Although no consensus has been reached regarding the origin of the different observed colors, partly due to the different defect configurations in different samples [3,38], ZnO provides the potential for creating white light emitting diodes, especially considering the recent progress in the growth and reproducibility of ZnO nanostructures grown on a variety of other p-type substrates [2]. The development of low temperature chemical growth approaches as suitable techniques for large area synthesis of ZnO nanorods with excellent luminescence properties on any substrate opens up new possibilities for developing hybrid ZnO pn junctions. One of these hybrid junctions is a combination of ZnO nanorods and p-type semiconducting polymers. Below, we will report recent results obtained from different ZnO nanorods and nanotubes grown through different methods on p-SiC, p-GaN, and p-polymer layers on flexible plastic, and we will report their use to fabricate white light emitting diodes (LEDs).

**Table 1:** Some recently reported lines emitted from ZnO and the proposed associated deep level defect(s) causing the emission. The conduction and valence bands are abbreviated in the usual way as C.B. and V.B., respectively.

Emission color (nm)	Proposed deep level transition
Violet	Zn_i_ to V.B. [3]
Blue	Zn_i_ to V_Zn_ or C.B. to V_Zn_ [3,70]
Green	C.B. to V_O_, or to V_Zn_, or C.B. to both V_O_ and V_Zn_ [67,32,33,34]
Yellow	C.B. to Li, or C.B. to O_i_ [9,35,36,37]
Orange	C.B. to O_i_ or Zn_i_ to O_i_ [3]
Red	Lattice disorder along the c-axis (*i.e.* due to Zn_i_) [41]

## 3. Results and Discussion

**Figure 2:**
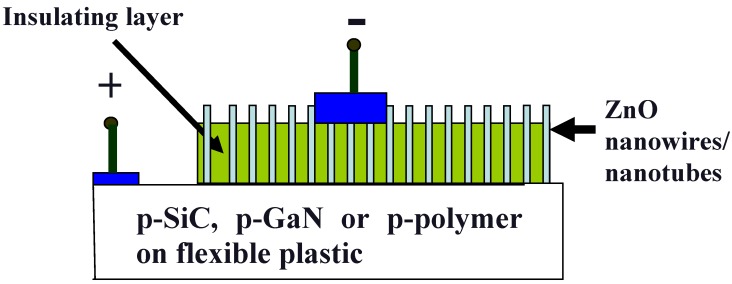
A schematic diagram of n-ZnO nanostructures on different p-type substrates.

This section presents some of our recent LEDs fabricated using ZnO nanorods and nanotubes grown by a high temperature evaporation method (vapor liquid solid technique) and by a low temperature method (aqueous chemical growth) at temperatures as low as 50 °C. Figure 2 presents a diagram showing the structure of all of the fabricated white LEDs. As shown in the figure, all white LEDs were fabricated with ZnO nanorods or nanotubes grown on top of the different p-type substrates.

### 3.1. n-ZnO nanorods/4H-p-SiC LEDs

**Figure 3:**
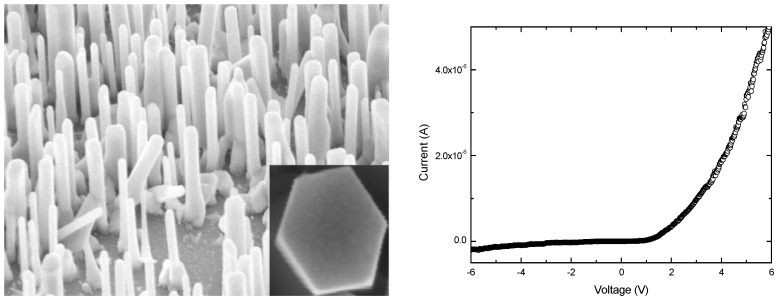
(Left) A typical SEM image of ZnO nanorods grown by the VLS technique on a 4H-p-SiC epitaxial layer. (Right) Typical room temperature I-V characteristics obtained from the LED fabricated with n-ZnO nanorods/4H-p-SiC.

ZnO nanorods were grown using the vapor liquid solid (VLS) high temperature technique on a 4H-p-SiC epitaxial layer, for the purpose of forming a pn heterojunction (described below). The grown nanorods were found to be aligned vertically and the nanorod length varied within the range 1.9–2.2 μm, as shown in the scanning electron microscope image (SEM) in Figure 3.

The optical properties of grown ZnO nanorods on the 4H-SiC sample were investigated using room temperature photoluminescence (PL), and the result (not shown here) indicates a sharp ultra-violet (UV) emission line around 380 nm due to the bandgap edge, as well as broad green luminescence bands centered around 530 nm and covering a large range of the visible spectrum. The observation of sharp UV emission indicates that the ZnO nanorods had a high crystal quality.

**Figure 4:**
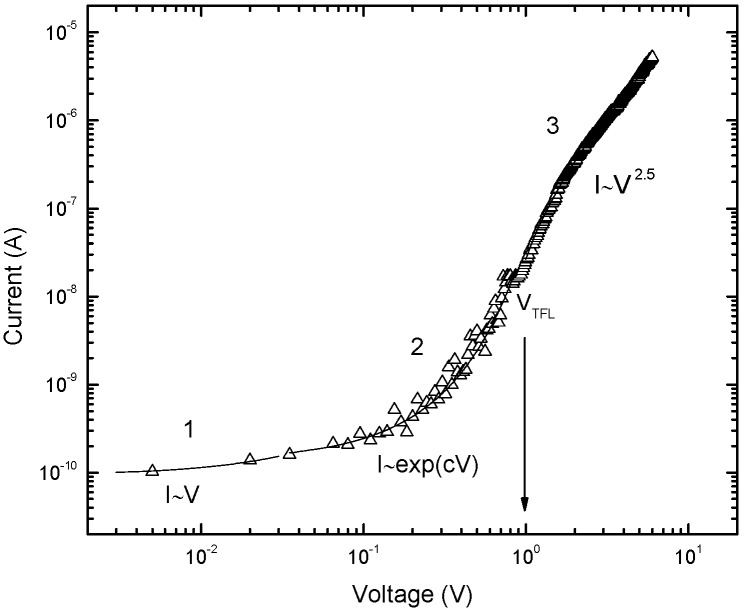
Log-log plot of the I-V characteristics of the 4H-p-SiC/n-ZnO heterojunction LED.

Figure 3 shows the resulting typical current-voltage characteristics of the ZnO nanorods/4H-p-SiC heterojunctions analyzed using standard thermoionic emission theory. According to this theory, the current in such a device can be expressed as

(2) $$I=I_{S}[\exp\left(\frac{qV}{nk_{B}T}\right)-1],$$

where I_s_ is the saturation current, k_B_ is the Boltzmann constant, T is the absolute temperature, q is the elementary electric charge, V is the applied voltage, and n is the ideality factor. The ideality factor in Eq. (2) was found to be in the range of 3–4 for the investigated diode. A higher ideality factor indicates that the transport mechanism is no longer dominated by thermionic emission. Non-ideal behavior is often attributed to variations in the interface composition and to other current transport mechanisms provided by other defect states in the band gap of the semiconductor such as structural defects, surface contamination, barrier tunneling, or generation and recombination in the space charge region [45,46]. To understand which mechanisms influence the junction behavior, the I-V characteristics of the device are studied on a log-log scale. A log-log plot of the I-V data at RT is shown in Figure 4, and it demonstrates that the current transport mechanism exhibits three different regions. The current in region 1 follows a linear dependence, *i.e.*, I~V. This indicates that transport is dominated by tunneling at low voltages. The boundary for this region was determined to be 0.03 V. In region 2 (0.04–1 V), the current increases exponentially, I~ exp (cV). The ideality factor (3–4) is determined in this region, and the dominant transport mechanism is recombination-tunneling. Finally, above 1 V (region 3) the current follows a power law (I~V^2.5^), indicating a space-charge limited transport mechanism. The space charge limited current (SCLC) region observed in the present study has also been reported in different n-ZnO nanorods/p-Si heterojunction LEDs [47,48,49].

Lampert and Mark [50] have developed the single carrier SCLC model in the presence of a trap above the Fermi level. According to this model, at an applied voltage of V>V_TFL_ (TFL indicates the trap filled limit) all of the trap levels are filled, the conduction becomes space charge limited, and the current follows the Mott and Gurney SCLC expression [51]. The trap filled limit voltage V_TFL_ is given as [50]:

(3) $$V_{TFL}=\frac{N_{t}qd^{2}}{2\varepsilon \varepsilon_{0}}$$

where N_t_ is the concentration of unoccupied states (trap concentration) located approximately at the estimated effective Fermi level. The effective carrier concentration n_o_ in the active region is given by the expression:

(4) $$\frac{J(2V_{TFL})}{J(V_{TFL})}\approx \frac{N_{t}}{n_{0}},$$

where J(V_TFL_) is the current density at V_TFL_ and J (2V_TFL_) is the current density at a voltage of twice V_TFL_. The position of the effective Fermi level (quasi-Fermi level) can be estimated from the calculated value of n_o_. Deep level parameters were calculated from the experimental V_TFL_ at RT. The depletion region thickness (1.2 µm) at zero bias capacitance was used as the active layer thickness in these calculations. The values of n_o_, N_t_, and the location of the deep level states (traps) below the conduction band were determined to be 3.4 × 10^17^ cm^-3^, 4.4 × 10^18^ cm^-3^, and ~0.24 ± 0.02 eV, respectively. These observed deep level states are in agreement with the reported data for the zinc interstitial (Zn_i_) level, which is theoretically located 0.22 eV below the conduction band [52]. Violet emission has recently been reported to originate from Zn_i_ [3]. In addition, according to calculations based on the full potential linear muffin-tin orbital method, the transition energy from the Zn_i_ level to the valence band in ZnO corresponds to 3.1 eV [53]. This agrees well with our experimental results. The extracted transition energy from the observed trap (Zn_i_) to the valence band is 3.13 ± 0.02 eV.

**Figure 5:**
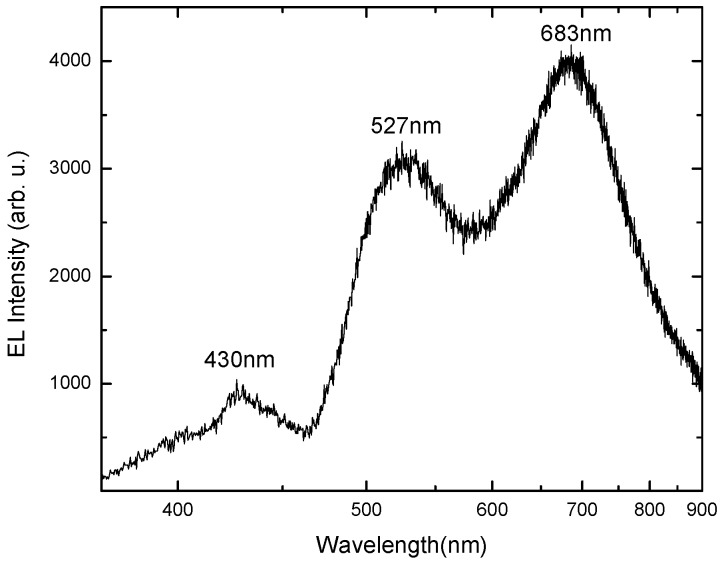
Room temperature EL spectrum of the 4H-p-SiC/n-ZnO heterojunction LED, revealing broad emission covering the entire visible spectrum.

The EL measurement of the ZnO-based heterojunction was carried out using a photomultiplier detector at room temperature. Figure 5 shows the corresponding EL spectrum, indicating three peaks at 430 nm (violet), 527 nm (green) and 683 nm (red). Several groups have reported that different defect centers in ZnO are responsible for the blue, green and red emissions [3,54]. Leiter *et al.* observed a broad green band centered at 2.45 eV and assigned it to the oxygen vacancy (Vo) [55]. The 2.38 eV green emission has been attributed to the oxygen antisite (O_Zn_) on theoretical grounds [56]. There is still controversy in the literature about the origin of the luminescence centers observed in ZnO materials [57]. Recent studies have demonstrated that the green emission at 533 nm is related to oxygen vacancies V_O_, and the red emission at 683 nm is related to zinc vacancies or excess oxygen [3,52]. Our EL spectra demonstrate peaks at 527 nm and 683 nm, and these are due to V_O_ and V_Zn_ in the ZnO nanorods, respectively [3,33]. The violet emission at 430 nm is due to the interstitial zinc (Zn_i_) [3]. It was recently reported that the violet emission corresponds to Zn_i_ and the transition involving V_Zn_ would result in blue emission [3,39,58]. It has also been reported that in wide band gap semiconductors, the broad band luminescence is related to transitions from donor states to deep acceptor states [59].

Cathodoluminescence (CL) spectroscopy was performed to obtain detailed emission information and to explain the origin of specific emissions from specific areas. The cathodoluminescence spectroscopy technique involves an incident electron beam of an energy that produces free electron-hole pair recombination across the bandgap or between deep levels and the band edges. The CL penetration depth increases with increasing beam energy, as determined by the energy-range relationships for energy loss within a solid [60,61]. CL signals from different depths within the bandgap of the material can be excited, and the average depth distribution of the luminescence can thus be determined. The acceleration voltage for this study was varied from 10 to 30 kV, which corresponds to maximum penetration depths of 0.4–2.16 µm, as calculated from the Kanaya-Okayama model [61].

**Figure 6:**
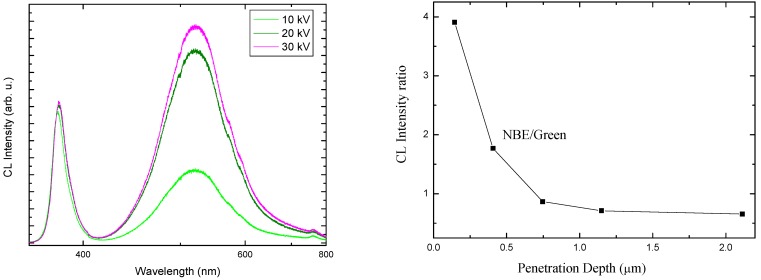
(left) Depth-dependent cathodoluminescence (CL) spectra of ZnO NRs, taken with a spot size of 50 nm at room temperature, at different accelerating voltages and a constant current of 56 pA. (right) The emission intensity ratio of the near band edge and green bands as a function of penetration depth.

To understand the emission properties of ZnO nanorods, it is necessary to clarify the effect of electron beam conditions on the CL measurement. The accelerating voltage is considered to be the main parameter that affects the electron beam [62]. Therefore, depth-dependent CL spectra were collected. Figure 6 (left) shows the RT-CL spectra at different accelerating voltages (10–30 kV) for a constant current of 56 pA. The number of excited carriers is assumed to be proportional to the accelerating voltage. The CL spectra exhibit NBE emission at 380 nm, which is related to the direct recombination of photon-generated charge carriers (excitonic emission), a green band centered at 524 nm, and red emission at 750 nm. Note that all of the CL spectra are red shifted because the sample was heated by electron bombardment.

The penetration depth, which is related to the rate of electron-hole pair creation, varies with the incident electron accelerating voltage, with values of 0.4, 1.14 and 2.16 μm for accelerating voltages of 10, 20 and 30 kV, respectively. Thus, an electron acceleration voltage of 20 kV corresponds to approximately 1 μm of penetration depth [60]. The RT-CL spectra exhibit a constant NBE as the accelerating voltage changes, while the green emission varies. This indicates that the native point defect concentration might vary with depth. When the accelerating voltage is increased, the electron beam is expected to penetrate deeper into the nanorods and excite more electron-hole pairs near and below the penetration depth. Because of this, more emission centers will be excited by the electron bombardment. In bulk ZnO and thin ZnO films, UV emission from ZnO can be internally reabsorbed by the crystal within a 1 μm range [63]. However, this reabsorbed UV emission can excite defect states in the material, resulting in the green emission. Thus, part of the UV emission may contribute to the enhancement of the green emission [64]. The differences and variations in the size of the nanostructures also contribute to the emission intensities. A luminescence spectrum usually represents the optical characteristics of all of the nanostructures inside the probed area, and due to inhomogeneities among the nanostructures, the spectrum can be considered an average luminescence [65]. Remarkably, the ratio of the NBE to the green intensity, which is used as an indicator of nanorod quality, decreases with penetration depth over the entire length of these nanorods. As a result, the right panel of Figure 6 indicates that there are more deep defects at the roots of the ZnO nanorods than in their upper parts. Willander *et al.* demonstrated through a HR-TEM study that ZnO nanorods have more structural defects at the interface between the ZnO nanorods and the substrate than at the top of the nanorod [2]. Chien-Lin *et al.* reported that the NBE and green are emitted separately from two opposite halves of the nano-rods [66]. CL spectroscopy gives in-depth information about radiative defects. Using this technique on our samples together with results from others [2,66] indicates that there are more deep defects (radiative defects) at the roots of the ZnO nanorods. Nevertheless, it has also been shown that depletion layers at surfaces or grain boundaries might alter the defect charge states and hence modify the resulting emission [67]. This effect can also contribute to the observed variation in green emission with depth [67]. To fully understand the origin of the correlation between luminescence and transport characteristics, more systematic investigation of the samples is necessary. The inhomogeneities observed among ZnO nanostructures can be attributed to fluctuations during the growth and formation of the nanostructures. Investigation of inhomogeneities among ZnO nanostructures grown by different methods can shed some light on the fundamental growth mechanisms and possible improvements.

### 3.2. n-ZnO nanotubes/p-GaN LEDs

**Figure 7:**
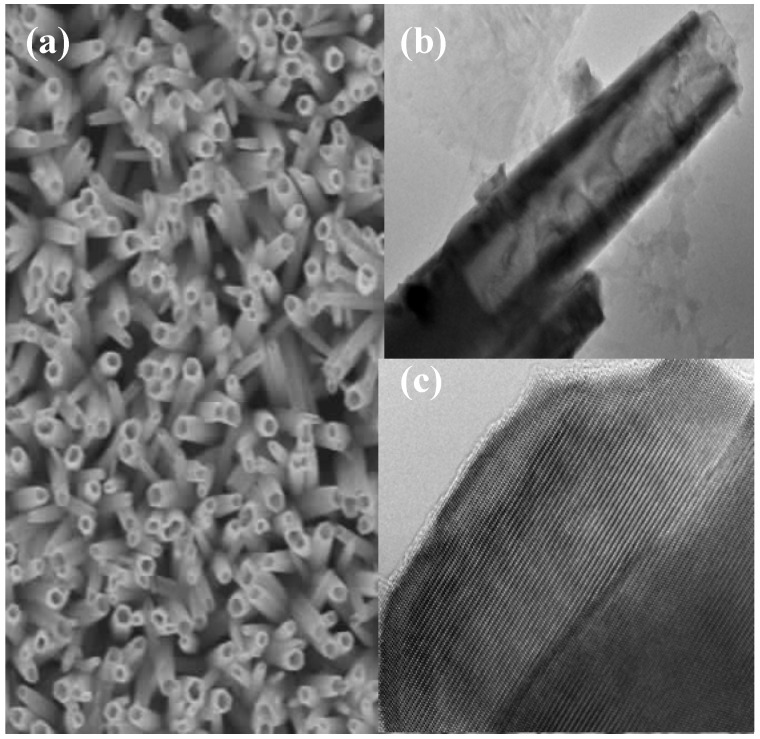
(a) SEM image of ZnO nanotubes grown on p-GaN. (b) Low resolution TEM image of a single nanotube, showing the depth of etching inside the nanotube. (c) High resolution TEM image of a single ZnO nanotube.

Nanotubes, or hollow nanorods, possess a much larger surface to volume ratio than nanorods. The increased surface area implies that the concentration of surface defects would be expected to increase. Surface states, which lead to deep radiative levels, can easily be manipulated by post-growth processing. This manipulation can lead to enhancement or changes in the nature of complex defects adsorbed to the surface, and hence, nanotubes add more degrees of freedom in LED design. Whispering gallery modes also may enhance the optical characteristics of ZnO nanotubes compared to nanorods [68]. Hence, the use of ZnO nanotubes will be expected to yield interesting results, with the possibility of manipulating the emission from surface defects. We have developed a technique to synthesize ZnO nanotubes with very high yield [68]. Figure 7(a) shows an SEM image of ZnO nanotubes with diameters between 100–150 nm, lengths from 1 to 2 microns, and wall thicknesses of 20–35 nm, showing epitaxial orientation almost normal to the substrate. It is clear from the image that these ZnO nanotubes are hollow with 100% yield. Transmission electron microscopy (TEM) was further used to confirm the tubular structure and to measure the etching depth of the ZnO nanotubes, which was intentionally kept within the range of 700–900 nm to protect the hetero-junction between the nanotubes and substrate, as seen in Figure 7(b). A high resolution TEM image (Figure 7(c)) shows that the ZnO nanotubes are single crystalline and the orientation of the nanotube in the microscope demonstrated that the growth direction of the ZnO nanotube is along the c-axis of the wurtzite structure. The etching mechanism is related to the wurtzite crystal structure of ZnO nanorods and relies on the difference in stability of the polar (0001) and non-polar (1010) planes. The structural stability of the polar planes of the nanorods is important for the high etching rate by the adsorption of chloride ions along the polar direction instead of the non-polar direction [69].

**Figure 8:**
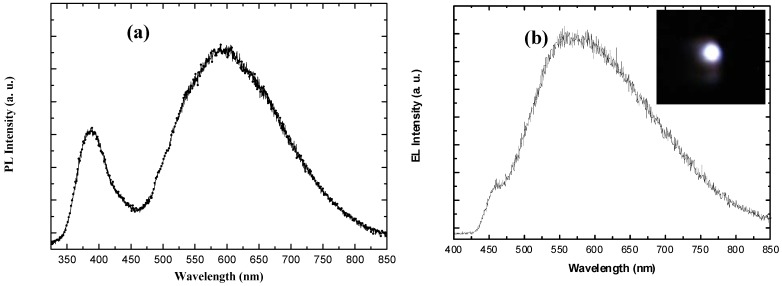
(a) Room temperature PL spectrum of ZnO nanotubes grown on GaN. (b) The corresponding electroluminescence spectrum obtained from the same ZnO nanotubes/p-GaN LED. The inset displays a digital photograph showing the light emission.

Figure 8(a) displays the room temperature photoluminescence (PL) of the ZnO nanotubes, revealing two emission bands: UV and visible. The former, relatively weak peak centered at 389 nm can be attributed to the radiative recombination of free excitons, while the visible broad band emission centered at 596 nm may be related to deep level defects. The reason for the broad visible band is that ZnO nanotubes have a large surface to volume ratio and a high porosity. The etching process used in nanorod fabrication is also an important source of surface defect formation. The deep level emission is known to be related to a variety of intrinsic (*V*_Zn_, *V*_O_, or Zn_i_) and extrinsic (acceptor) defects [6]. The electroluminescence (EL) characteristics of the n-ZnO nanotubes/p-GaN heterostructure LEDs were studied under a forward bias current.

The EL spectrum shows a large broad-band emission and relatively low blue and violet emission peaks centered around 570 nm and 450 nm, respectively, as shown in Figure 8(b). The emission over the broad band covering 475–800 nm is attributed to the injection of the holes from p-GaN toward the ZnO nanotubes and their recombination with defect states in the ZnO nanotubes. The injected forward current activates the more radiative recombination centers in ZnO nanotubes, which enhances the brightness of the white light emission, along with the large number of surface defect states and bulk defects in the ZnO nanotubes [68]. The origin of the blue-violet peak centered at 450 nm is still controversial, with possible sources of the transition from zinc interstitial to zinc vacancy [3,70] and the radiative recombination related to deep Mg acceptor levels [71,72].

### 3.3. n-ZnO nanorods/p-polymer hybrid LEDs

As mentioned above, ZnO possesses the property of self-organized growth. This property has made it possible to grow ZnO nanostructures with device-quality crystals on a variety of substrates, even those of an amorphous nature [2]. Moreover, the possibility of low temperature aqueous chemical growth enables the growth of ZnO nanorods with excellent luminescent properties on large area substrates. This possibility suggests the combination of ZnO nanorods and organic polymer electrodes to form a hybrid lighting technology that avoids the problems associated with p-type ZnO. These hybrid organic-inorganic light emitting diodes have recently been suggested, and the electroluminescence of these materials has been reported by different groups [73,74,75,76,77]. In our previously presented organic-ZnO nanorod hybrid light emitting diodes, we grew the nanorods on glass substrates at a temperature of 95 °C [75,76]. PEDOT:PSS was first spin-coated onto the glass and flowed by the p-type-polymer(s) layer(s). The output emission intensity and wavelength of these materials can be modified by engineering the p-type polymer layer sequence. We have designed different multi-layer p-type polymers with the strategy of having a divided hole barrier to improve the current and hence increase the output intensity of light emitted from this organic-inorganic hybrid structure [75]. The hole transport in the organic electrode and the viscosity of the polymer were considered, and pure and blended configurations were investigated [75]. We also demonstrated that it was possible to optimize the electrical and electro-optical emissions of this hybrid LED by inserting a proper hole barrier divider between the p-type polymer and the ZnO nanorods [76]. Here, we present our most recent results from an even lower temperature hybrid LED on flexible plastic. We have been able to grow ZnO with good structural and excellent luminescent properties at 50 °C on flexible plastic, and this material emits white luminescence as shown below.

**Figure 9:**
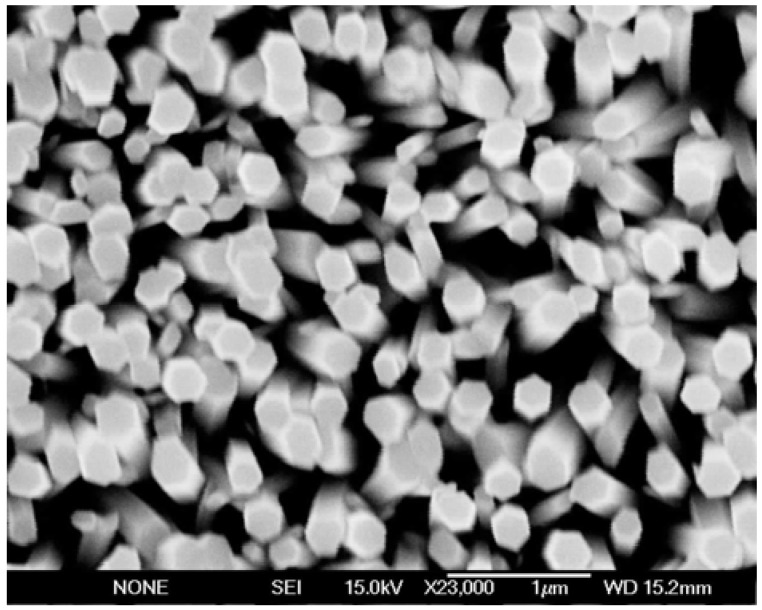
Typical SEM image showing ZnO nanorods grown at 50 °C. These ZnO nanorods were grown on a flexible plastic substrate using the aqueous chemical approach.

**Figure 10:**
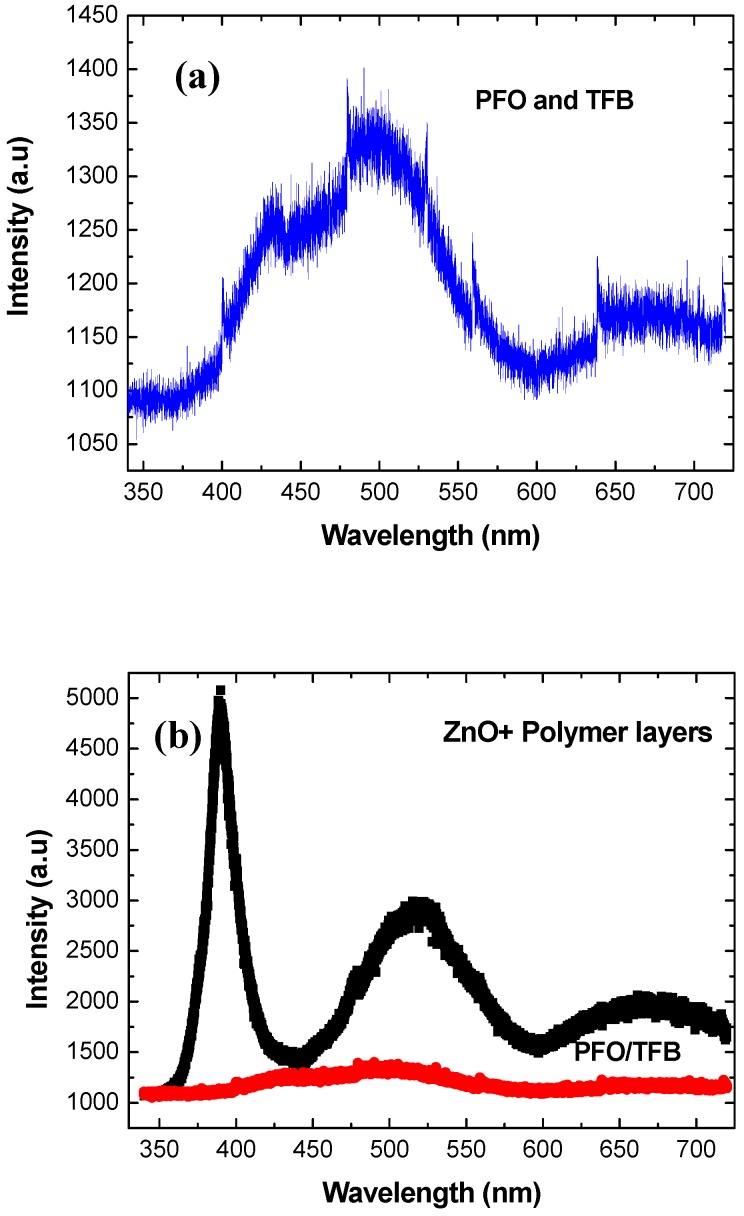
(a) Room temperature PL spectrum obtained from p-type polymer(s) layers spin-coated onto flexible plastic. (b) The corresponding room temperature PL spectrum after the low-temperature growth of ZnO nanorods. The lower curve represents the PL presented in part (a) of the figure to allow for a comparison of intensities.

Growth of ZnO nanorods on flexible substrates was performed at temperatures as low as 50 °C; the details of the growth procedures will be discussed elsewhere. The as-deposited ZnO nanorods demonstrate a c-axis preferential orientation, as can be seen in the SEM micrograph in Figure 9. This image clearly shows well-aligned nanorods with hexagonal tips, diameters of about 200 nm and an average length of about 1.2 μm. X-ray diffraction analysis revealed that c-axially oriented ZnO nanorods were achieved because the 002 peak dominated the XRD spectrum (not shown here). ZnO nanorods grown at 50 °C were combined with p-type polymers to fabricate hybrid LEDs. Before the processing of the device, room temperature PL was performed on the polymers on the flexible substrate and was performed again after the low temperature growth of the ZnO nanorods. The results are shown in Figure 10(a-b). The device was constructed using the well-known PFO p-type polymer, a blue light emitter. The polymer layer sequence was as follows: first PEDOT:PSS was deposited onto the flexible plastic, then TFB was spin-coated, followed by the PFO. The TFB was inserted to divide the relatively large hole barrier between the PFO and the PEDOT:PSS. Figure 10a shows the room temperature PL spectrum of the PFO/TFB polymer multi-layers spin-coated onto the flexible substrate. As expected, blue emission due to the PFO is observed in the PL spectrum, as shown in Figure 10a. Figure 10b shows the PL of the ZnO nanorods grown on top of the polymer layers on flexible plastic. As clearly indicated by the PL, intense near band emission band at 390 nm demonstrates radiative recombination between electrons from the ZnO conduction band with holes in the valence band or a defect level close to it. Moreover, defect-related transitions are apparently observed in the PL spectrum, where a broad band around 520 nm (green band) as well as a third broad peak at 660 nm (orange red band) were both observed. The origin of these defects’ radiative transitions was discussed above; they can be attributed to oxygen and zinc vacancies and oxygen interstitial atoms, respectively [3,33]. The as-deposited ZnO nanorods grown at low temperature on a flexible substrate were then used to fabricate an LED.

**Figure 11:**
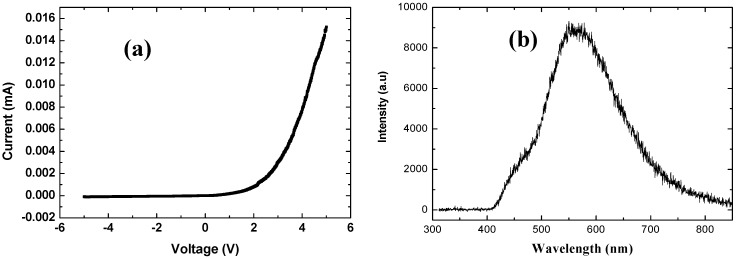
(a) I-V characteristics of the hybrid LED obtained using ZnO nanorods grown at 50 °C. (b) Electroluminence spectrum obtained from the hybrid LED revealing white luminescence covering the entire visible range (from above 400 nm and up to nearly 700 nm), resulting from the combination of the PFO/TFB and the low-temperature grown ZnO nanorods. The PFO emission at 450 nm appears in the EL as a shoulder.

The electrical behavior of the hybrid LEDs obtained using the ZnO nanorods grown at 50 °C was investigated, and rectifying behavior was observed. Figure 11(a) shows typical I-V characteristics, revealing rectification behavior with a turn-on voltage as low as 2.4 V. Light emission was detected at current levels of about 0.2 mA. The diode was stored for a few weeks (12 weeks) and investigated again, and no degradation was observed. Figure 11(b) shows the electroluminescence spectrum of this hybrid organic-inorganic LED, with a broad band covering emissions from just above 400 nm and up to nearly 700 nm, with a peak at around 560 nm. As the next step in the construction of a functional LED, we are now examining the light quality and output power of this hybrid LED.

### 3.4. n-ZnO nanorods and external p-type electrodes

Although ZnO has been studied for many decades now, no commercial electro-optical components (LEDs or lasers) have been developed utilizing this excellent luminescent material. The main reason for this is the lack of a stable, reproducible p-type doping scenario for ZnO material. Many attempts have been made to achieve hetero-epitaxy of ZnO thin films on different p-type substrates. Nevertheless, none of these attempts have resulted in device-quality ZnO thin film/p-type heterostructures. As mentioned above, ZnO possesses the property of self-organized growth, which is beneficial for the growth of ZnO nanostructures. According to results published during recent years and shown above, ZnO nanostructures, especially nanorods and nanotubes can easily be grown on a variety of substrates [2,78]. Crystalline as well as amorphous substrates have been demonstrated to be good platforms for the growth of device-quality ZnO nanorods/nanotubes that form high-quality heterojunctions with different p-type substrates. In addition, these ZnO nanorods-nanotubes/p-type substrates have shown to yield white electroluminescence due to emission from the native deep levels that emit over the entire visible range as discussed above. With the use of techniques like aqueous chemical growth (see below), the large area growth of ZnO nano-structures can easily be achieved on a variety of substrates. The use of aqueous chemical growth of ZnO nanorods on a p-type polymer on flexible plastic substrates seems to be the best choice for the realization of commercial white light emitting diodes due to the cost and ease of use of large area substrates, and the possibility of integrating the flexible plastic with available lighting armature technology (glass bulbs and tubes). However, the growth parameters must first be optimized to obtain white light with a high color rendering index (CRI) and an acceptable light output efficiency (50 lumen/Watt). Moreover, the long-term stability must be investigated before these commercial products can be made available.

## 4. Experimental Section

The results presented above in Section 3 were achieved using ZnO nanorods/nanotubes synthesized using two different techniques, the vapor liquid solid (VLS) technique performed at high temperatures (> 850 °C) and low-temperature (<100 °C) aqueous chemical growth (ACG). Below, we explain these two techniques and briefly describe the processing of the presented LEDs.

**Figure 12:**
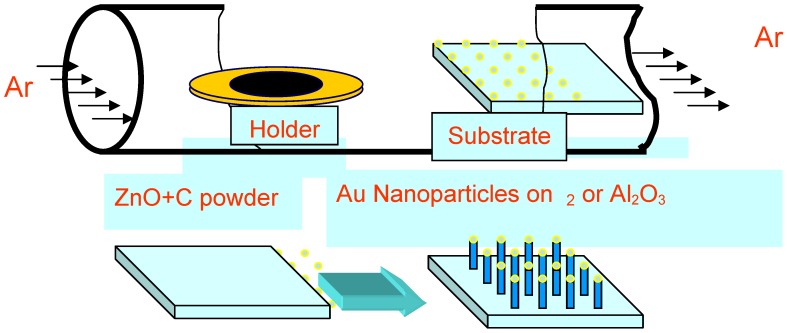
Schematic diagram showing the quartz tube furnace typically used for the vapor liquid solid growth of ZnO nanorods.

VLS based processes have been widely used for the growth of various nanostructures since the first demonstration in 1964 by Wagner and Ellis [79]. The VLS process can be divided into two steps: the formation of a small liquid droplet and the nucleation and growth of the nanorods. Generally, the growth of ZnO nanorods is accomplished by employing metal clusters as catalysts. The metal can be chosen rationally using the phase diagram on the basis of the solubility of the nanorods’ component elements in the liquid phase. The growth direction and diameter of the ZnO nanorods during the growth process are highly dependent on the metal droplet, which is composed of the metal catalyst and the nanorod material. Au is the most commonly used catalyst for the growth of ZnO nanorods when using the VLS growth method [80] because Au has the ability to form a eutectic mixture with the ZnO material at temperatures far below the melting point of ZnO. Other metals such as tin have also been successfully used to synthesize high-quality ZnO nanorods [5]. The method of thermal evaporation was used to grow ZnO nanorods on a p-SiC substrate using Au as a catalyst in a flat quartz oven. Figure 12 shows a typical experimental setup used for the growth of ZnO nanorods. A thin layer of Au (3–5 nm) was first deposited onto the p-SiC substrate. High-purity (99.9%) commercial ZnO powder was used as the main source material. The high purity ZnO powder was mixed with high purity (99.9%) graphite at a 1:1 ratio, and the mixture was placed in a quartz boat. The mixed powder was vaporized at elevated temperatures (>890 °C) and Zn condensed onto the substrate containing the Au particles and then reacted with oxygen, resulting in catalyzed epitaxial growth of ZnO nanorods. The gas composition in the quartz furnace tube has a strong influence on the formation of ZnO nanorods. The diameter and size of the ZnO nanorods can be tuned by controlling the thickness of the evaporated Au and the growth time, respectively. A more detailed description of our VLS processes can be found in [81].

The ACG method mainly followed the method described in [82]. The ZnO nanorods were grown using zinc nitrate hexahydrate [Zn(NO_3_))_2_.6H_2_O, 99.9% purity] and methamine (C_6_H_12_N_4_, 99.9% purity) as precursors. Before growth, the substrate was pre-treated by forming a seed layer as described in [83]. The pre-treated substrate was immersed into the aqueous solution and tilted against the wall of the beaker. The beaker was then placed into a pre-heated oven at 93 °C for five hours. The beaker was removed from the oven and cooled to room temperature, followed by washing with de-ionized water to remove any residual precursors. The detailed growth procedure using the ACG method can be found in [83,84]. The ZnO nanotubes were obtained using ACG-grown ZnO nanorods by immersing the nanorods in an aqueous solution of potassium chloride (KCl) for seven hours at 95 °C. The parameters were optimized, e.g., nanorod morphology, immersion time, KCl concentration, etching temperature, *etc.*, and these optimized parameters were used to fabricate the nanotubes to the required depth [68].

All LEDs were processed using the same steps. The only difference was in the ohmic contact to the substrate. Depending on the substrate material (p-SiC, p-GaN, or p-polymer), the appropriate metal was first deposited onto a small part of the substrate; this was followed by the ACG growth method. This step was performed after growth when using the VLS growth technique due to the high temperature during growth. In the case of p-SiC and p-GaN, the metal deposition was followed by annealing to form an intimate contact with low contact resistivity. The part of the substrate that was covered with the ohmic contact was protected and the growth was then performed as described above. Before forming the ZnO nanorod ohmic contact, an insulating layer was deposited to avoid shortage between the top ZnO ohmic contact and the substrate. To achieve this insulation, a photoresist was spin-coated onto the ZnO nanorods after protecting the area used for ohmic contact with the substrate. After spinning the photoresist, low power oxygen plasma reactive ion etching was performed. This step was used to expose a small part of the nanorods to form the ZnO ohmic contact. Aluminum followed by platinum was used as the top non-alloyed ohmic contact to ZnO.

## 5. Conclusions

This paper discussed ZnO deep level centers and the origin of the broad band(s) related to defect emission based on recently published results. Although there has been no consensus on the origin of the visible deep level emission bands in ZnO, recent results indicate that the broad green emission band might have multiple sources. Recent comparative studies on samples grown by different techniques show that although a broad defect band is present, the center of the broad defect band is different in different samples. This is due to the fact that different samples will contain different concentrations of deep levels and other complexes due to the different growth environments. Samples grown in Zn-rich or O-rich environments will both show broad bands, but they will be centered at different wavelengths. Despite the fact that no consensus has been reached, ZnO emits all of the visible colors due to deep level centers. With proper optimization of the growth conditions, it is possible to reproduce ZnO nanorods with the same luminescence properties. Because ZnO possesses the property of self-organization combined with the small footprint of nanorods, ZnO nanorods can be grown on a variety of p-type substrates, avoiding the difficulty of doping ZnO to p-type. We have demonstrated different white light emitting diodes based on ZnO nanorods and crystalline p-type semiconductors, including 4H-p-SiC and p-GaN. Moreover, ZnO nanotubes with a much larger surface area to volume ratio were employed to fabricate ZnO-based LEDs. Using ZnO nanotubes resulted in a broader spectrum, leading to improvement of the white light quality. This is probably due to the enhanced deep level defects associated with complexes adsorbed at the surface of the nanotube. Growth at temperatures as low as 50 °C has been demonstrated and shown to yield ZnO nanorods with excellent visible luminescence. This low growth temperature allows the combination of luminescent n-ZnO nanorods and p-type polymers on flexible plastic. This hybrid technology could lead to the development of large-area white lighting technology.
